# The Role of the Autonomic Nervous System in the Regulation of Aortic Stiffness

**DOI:** 10.1161/HYPERTENSIONAHA.116.08035

**Published:** 2016-10-12

**Authors:** Kaisa M. Mäki-Petäjä, Sharon M.L. Barrett, Sarah V. Evans, Joseph Cheriyan, Carmel M. McEniery, Ian B. Wilkinson

**Affiliations:** From the Division of Experimental Medicine and Immunotherapeutics, University of Cambridge, United Kingdom.

**Keywords:** aortic stiffness, autonomic nervous system, blood pressure, healthy volunteers, heart rate, hemodynamics

## Abstract

The autonomic nervous system is important in regulating blood pressure, but whether it regulates aortic stiffness is more contentious. We conducted 3 studies in young, healthy individuals to address this important question. Study 1 was a cross-sectional study of 347 subjects with detailed measurements of hemodynamics and heart rate variability. In study 2, 9 subjects were given a bolus of intravenous nicotinic ganglion blocker, pentolinium, or saline in a random order and hemodynamics and heart rate variability were assessed before and after. In study 3, changes in hemodynamics and heart rate variability were assessed during stimulation of the sympathetic nervous system with the use of isometric handgrip exercise in 12 subjects. Study 1: aortic pulse wave velocity (*P*=0.003) was lowest in the subjects with the highest parasympathetic activity, but after adjusting for mean arterial pressure, the effect was abolished (*P*=0.3). Study 2: after pentolinium, sympathetic and parasympathetic activity fell (*P*=0.001 for both), mean arterial pressure, and heart rate increased (*P*=0.004 and *P*=0.04, respectively), but there was no change in pulse wave velocity in comparison to placebo (*P*=0.1). Study 3: during handgrip exercise, sympathetic activity (*P*=0.003), mean arterial pressure (*P*<0.0001), and aortic pulse wave velocity increased (*P*=0.013). However, pulse wave velocity adjusted for mean arterial pressure did not change (*P*=0.1). The main finding of these studies is that in young healthy subjects, the autonomic nervous system does not have a pressure-independent role in the regulation of aortic stiffness. However, these findings may not apply to patients with increased sympathetic tone or hypertension.

The autonomic nervous system (ANS) has an important role in regulating blood pressure (BP) via autonomic vasomotor nerves and circulating catecholamines. Traditionally, increased sympathetic nervous system (SNS) activity was thought to be involved with the short-term regulation of BP, but it is now understood that the ANS also has an important role in the long-term regulation of BP, and that changes in ANS activity may be responsible for so-called idiopathic hypertension.^1^ Indeed, hypertensive patients have increased sympathetic and reduced parasympathetic activity in comparison to normotensive controls.^2^

Whether the ANS regulates aortic stiffness, an important independent predictor of fatal and nonfatal cardiovascular events,^3^ is more contentious. The main regulators of aortic stiffness are thought to be structural components of the arterial wall, distending pressure, and smooth muscle. Vascular smooth muscle tone is, in part, regulated by sympathetic neural activity via release of the potent vasoconstrictor noradrenaline. Conversely, most arteries in the body do not have parasympathetic innervation. Animal and human data indicate that the ANS modifies the stiffness of muscular brachial and carotid arteries.^4–6^

In contrast in vitro experiments in the human aorta have not shown any effect of the ANS.^7^ The results of human in vivo studies looking into the relationship between SNS activity and aortic stiffness are also conflicting with some concluding that there is a relationship^8,9^ and others not.^10,11^ Many studies have also failed to adjust for the changes in mean arterial pressure (MAP) and heart rate (HR),^12,13^ making it difficult to interpret the results.

To investigate the role of the ANS in the regulation of aortic stiffness, we conducted 2 studies: (1) a large cross-sectional study of ANS activity and aortic stiffness in young healthy individuals; (2) an intervention study in which ANS activity was suppressed by a ganglion blocker, pentolinium; and (3) an intervention study in which the SNS activity was stimulated via isometric handgrip exercise.

## Methods

### Study 1

#### Study Population

The Enigma Study is a long-term follow-up study of young individuals, investigating the origins of hypertension with regard to clinical, physiological, and genetic characteristics.^14^ Heart rate variability (HRV) measurements were made in a subset of individuals. Subjects with hypertension (BP >140/90 mm Hg) diabetes mellitus, a serum cholesterol level of 6.5 mmol/L, renal disease, cardiovascular disease, and those receiving any medication were excluded, leaving 347 individuals for the present analyses.

#### Hemodynamic Measurements

All studies were conducted in a quiet, temperature-controlled room. After 15 minutes of supine rest, BP was recorded in the brachial artery using a validated oscillometric technique (HEM-705CP; Omron Corp, Kyoto, Japan).^15^ Radial artery waveforms were obtained with a high-fidelity micromanometer (SPC-301; Millar Instruments, Houston, TX) from the wrist, and a corresponding central waveform was generated using a validated transfer function (SphygmoCor; AtCor Medical, Sydney, Australia).^16^ Augmentation index, a composite measure of wave reflection, MAP, and HR were determined using the integrated software. Aortic pulse wave velocity (aPWV) was measured as previously described.^17^

ANS activity was assessed by taking a 10-minute recording of HRV with the SphygmoCor device. The software calculates the statistical parameters of the normal R–R intervals (N–N intervals) by excluding ectopic beats of the ECG, thus allowing the analysis of both time and frequency domains. The time–domain indices included in our analysis were SDNN (SD of the R–R intervals), as a measure of total HRV; RMSSD (root mean square difference of successive normal R–R interval); and pNN50 (number of pairs of N–N intervals differing by more than 50 ms divided by the total number of N–N intervals in the entire recording), both measures of parasympathetic autonomic function. The frequency-domain parameters that were included were the power in the low-frequency (LF) range, representing mostly sympathetic activity; power in the high-frequency (HF) range, representing parasympathetic function and total power of all frequencies.

Cardiac output (CO) was assessed using a noninvasive, inert gas rebreathing technique.^18^ Briefly, while resting, subjects continuously rebreathed a gas mixture (1% SF_6_, 5% N_2_O, and 94% O_2_) over 20 s, with a breathing rate of 15/min. Expired gases were sampled continuously and analyzed by an infrared photoacoustic gas analyzer (Innocor; Innovision A/S) for the determination of CO and stroke volume (SV). Peripheral vascular resistance (dynes/s per cm^−^^5^) was calculated using formula peripheral vascular resistance=80×(MAP/CO).

### Study 2

#### Study Population

Nine healthy volunteers, aged between 20 and 34 years were studied. All participants reported that they undertook regular physical activity at least once a week. All experiments were conducted in the Vascular Research Clinics at Addenbrooke’s Hospital, Cambridge, United Kingdom. Subjects with chronic medical conditions, as defined for study 1 were excluded. Subjects were asked to abstain from alcohol and caffeine for 24 hours before study visits and to fast for 4 hours on the morning of the study.

#### Study Protocol

This was a double-blinded, placebo-controlled, randomized, cross-over study. Volunteers made 2 visits to the unit, separated by 1 week. At the beginning of each visit, a 21 to 23 g of cannula was inserted to the nondominant arm at the antecubital fossa. After 15-minute rest, baseline measurements of BP, MAP, HR, aPWV, CO, and SV and a recording of HRV were performed. After the baseline measurements, an intravenous bolus of pentolinium (BCM Special, Nottingham, United Kingdom) 5 mg/5 mL or saline 5 mL were given in random order. All hemodynamic measurements were repeated at 5, 10, 15, 20, and 25 minutes after the bolus of pentolinium/saline. Timings were based on our pilot data, in which we found that the maximal effect of pentolinium on HRV parameters was observed at 20 minutes after administration of the drug.

#### Hemodynamic Measurements

Hemodynamic measurements were performed as described for study 1, except that CO and SV were assessed noninvasively using transthoracic bioimpedance (CardioDynamics, BioZ Electrical) via electrodes attached at the neck and chest; and ANS activity was assessed by recording a 5-minute HRV with the SphygmoCor system.

### Study 3

#### Study Population

Twelve healthy volunteers, aged between 21 and 42 years were studied. All participants reported that they undertook regular physical activity at least once a week. All experiments were conducted in the Vascular Research Clinics at Addenbrooke’s Hospital, Cambridge, United kingdom. Subjects with chronic medical conditions as defined for the study 1 were excluded.

#### Study Protocol

This was an open-label observational study. Volunteers made a single visit to the Vascular Research Clinic. After 15-minute supine rest, baseline measurements of BP, MAP, HR, aPWV, and HRV were performed. After the baseline measurements, subjects were asked to perform their maximal voluntary contraction with a hand grip strength dynamometer (Takei Scientific Instruments Co, Tokyo, Japan). To stimulate SNS activity, subjects were asked to grip the dynamometer at 30% of their maximal voluntary contraction^19^ for a 4-minute period during which all hemodynamic measurements and HRV were repeated.

#### Hemodynamic Measurements

Hemodynamic measurements were performed as described for study 1, except that CO and SV were not assessed and ANS activity was assessed by recording HRV for 4 minutes with the SphygmoCor system.

## Data Analysis

### Study 1

Subjects were divided into tertiles of parasympathetic activity, based on the HF power. The primary outcome of study 1 was the difference in aPWV between tertiles of HF power. Secondary outcomes were the difference in BP, HR, CO, SV, peripheral vascular resistance, and HRV components between the tertiles and the relationship between HRV components and hemodynamic parameters.

After normality testing of the data with Kolmogorov–Smirnov test, histograms and Q–Q plots, the parameters that had either skew or kurtosis in the distribution were log-transformed for the subsequent analysis. The differences between the tertiles were analyzed using 1-way ANOVA with post hoc tests, where paired Student *t* tests with Bonferroni adjustment were used for multiple comparisons. Univariate analysis of variance was used to adjust PWV for MAP. Multiple regression analysis was used to explore the relationship between parameters.

### Study 2

Two-way repeated measures ANOVA was used to investigate the effect of the treatment. In post hoc tests, the effect of individual treatments was determined using paired Student *t* tests with Bonferroni adjustment. For the skewed variables, log-transformed values were used for the analyses.

### Study 3

The effect of hand grip exercise on hemodynamics and HRV were determined using paired Student *t* test. For the skewed variables, log-transformed values were used for the analyses. Univariate analysis of variance was performed to determine MAP-independent change in aPWV during the isometric handgrip exercise.

For all 3 studies, a probability of <0.05 was considered significant. Data are given as means±SD, unless stated otherwise. Approval for the studies was obtained from the Local Research Ethics Committee, and written informed consent obtained was taken from each participant. The studies were carried out in accordance with institutional guidelines and the Declaration of Helsinki.

## Results

### Study 1

**Figure 1.**
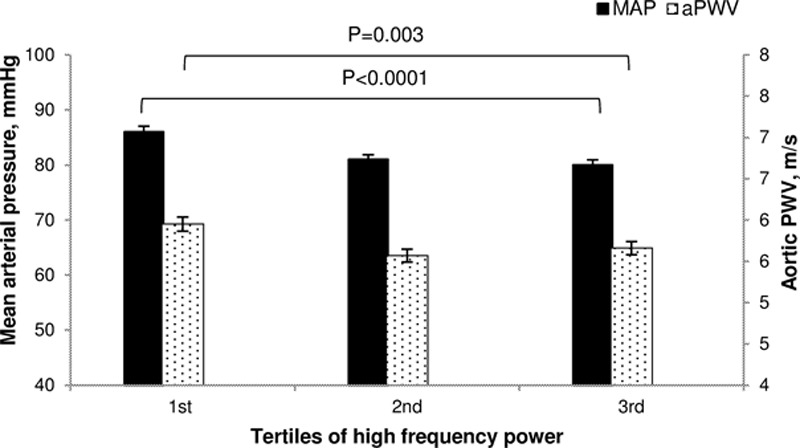
Mean arterial pressure (MAP) and aortic pulse wave velocity in tertiles of high-frequency power. Data represent means and SEM. Significance was determined using 1-way ANOVA with Bonferroni-corrected post hoc tests. n=347. MAP *P*<0.0001 between groups; post hoc tests: first vs second tertile: *P*<0.0001; second vs third tertile: *P*=0.6; first vs third tertile: *P*<0.0001. Aortic pulse wave velocity (aPWV) *P*=0.003 between groups; post hoc tests: first vs second tertile: *P*=0.004; second vs third tertile: *P*=0.4; first vs third tertile: *P*=0.03.

A total of 347 subjects were studied. The mean age of the subjects was 24±5 years, and 194 were female and 153 were male. The mean BMI was 23.3±3.2 kg/m^2^ and the mean BP was 119±16/74±10 mm Hg. The subjects were divided into tertiles according the HF power. Detailed baseline characteristics and hemodynamic parameters per tertile can be seen in Table 1. MAP reduced as the HF power increased (Figure 1; *P*<0.0001 for the trend), with the biggest difference in MAP seen between the first and third tertiles of HF power (86±11 versus 80±10 mm Hg; *P*<0.0001). Similarly, HR and CO were lowest in the third tertile of HF power. aPWV also reduced because the HF power increased (Figure 1; *P*=0.003 for the trend), with the largest difference seen between the first and second tertiles of HF power (5.95±0.94 versus 5.57±0.83 m/s; *P*=0.004). However, when aPWV was adjusted for MAP in a univariate analysis of variance, the difference in aPWV between the tertiles of HF power was abolished (*P*=0.3). Interestingly, when we repeated the analysis based on LF tertiles, we saw no difference in MAP (*P*=0.06) or aPWV (*P*=0.09) between the tertiles (data not shown).

**Table 1.**
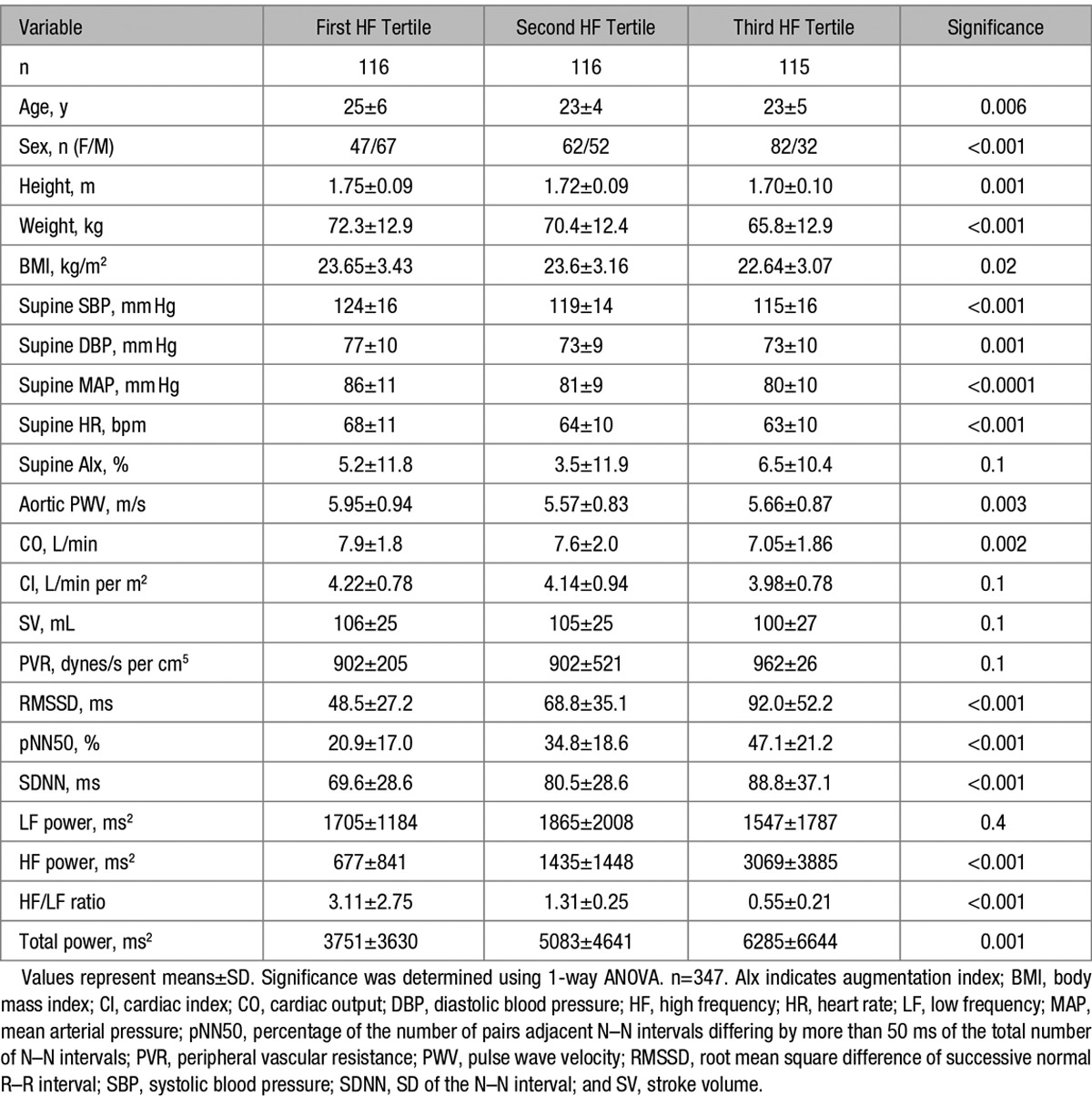
Demographic, Hemodynamic, and HR Variability Parameters per Tertiles of High-Frequency Power

In a stepwise multiple regression analysis for aPWV (Table 2A), we found that MAP, age, sex, and HR were independently associated with aPWV, whereas HF power failed to enter the model. In a stepwise multiple regression analysis for MAP (Table 2B), we found that BMI, HR, and age were independently associated with MAP, whereas HF power and sex failed to enter the model. In separate regression models (data not shown), a similar pattern was repeated if RMSSD, SDNN, pNN50, LF power, total power, and LF/HF ratio were entered into the model, instead of HF power, demonstrating that none of the HRV parameters were found to be independently associated with aPWV or MAP. Finally, in a stepwise multiple regression analysis for HR (Table 2C), we found that PNN50% (reflecting parasympathetic activity), sex, age, and BMI were independently associated with HR. In separate regression models (data not shown), we found that other HRV parameters (HF power, LF power, LF/HF ratio, total power, SDNN, and RMSSD) were also independently associated with HR after adjusting for age, sex, and BMI, but the strongest model was found when pNN50 was used as a measure of ANS activity (*R*^2^ for the model=0.391; *P*<0.0001).

**Table 2.**
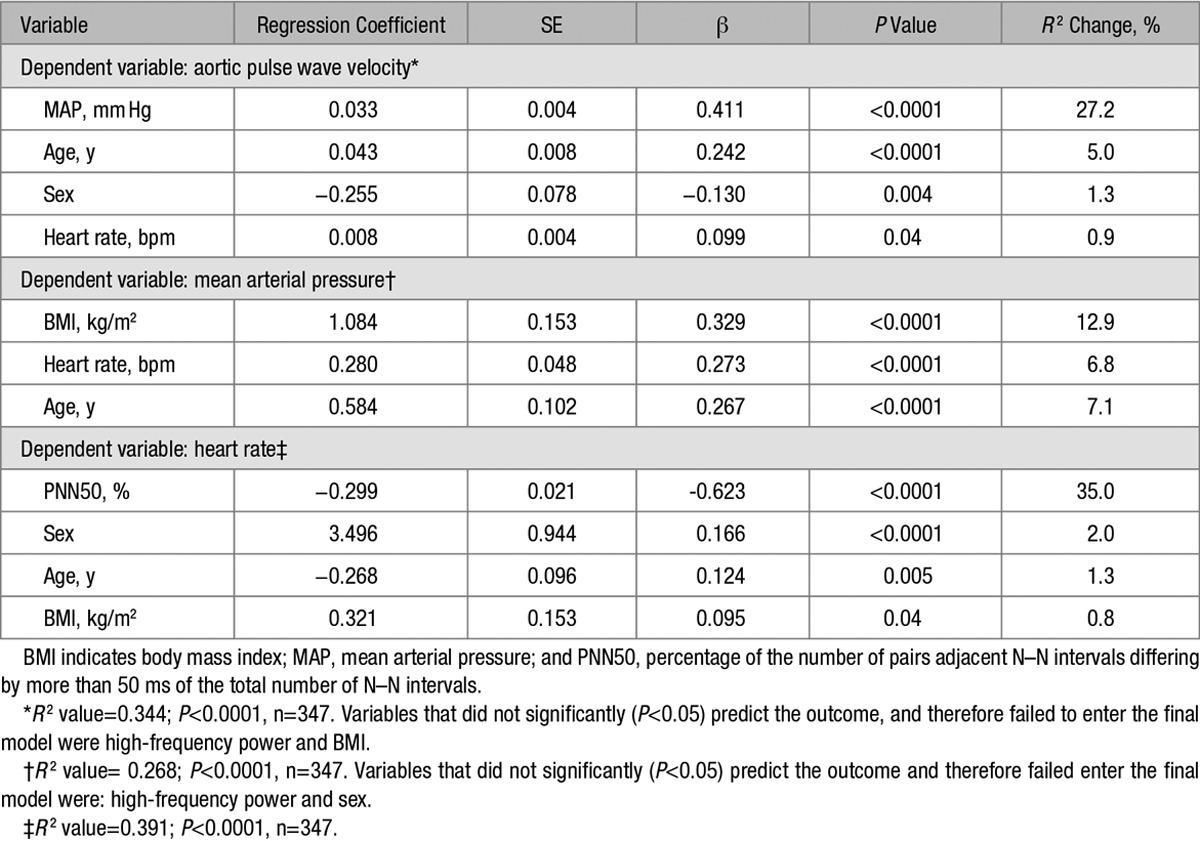
Stepwise Regression Analyses

### Study 2

**Figure 2.**
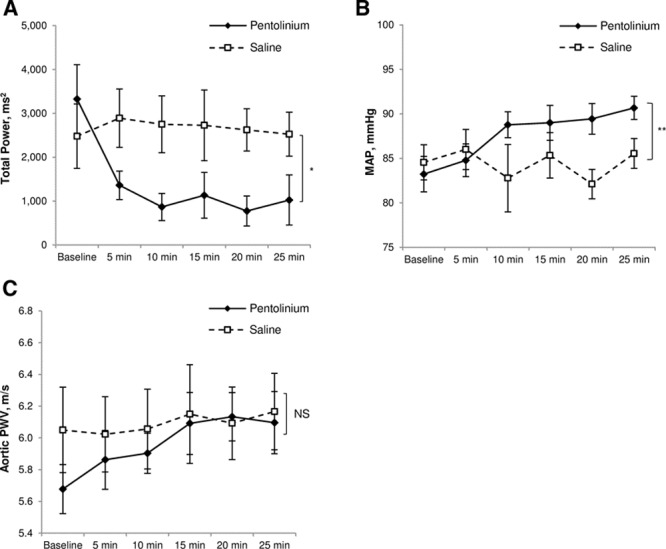
The effect of pentolinium and placebo on heart rate variability, blood pressure, and aortic pulse wave velocity. Data represent means and SEM. Significance was determined using 2-way repeated measures ANOVA with Bonferroni-corrected post hoc tests. n=9. **A**, Total power *P*=0.001 (*) between groups. **B**, Mean arterial pressure (MAP) *P*=0.004 (**) between groups. **C**, Aortic pulse wave velocity *P*=0.1 between groups. PWV indicates pulse wave velocity.

Nine subjects were studied in total. The mean age of the subjects was 28±6 years, and 4 were female and 5 were male. The mean BMI was 23.0±2.0 kg/m^2^ and BP was 118±8/69±11 mm Hg. The detailed effects of pentolinium and placebo on HRV and hemodynamics at baseline, 5, 10, 15, 20, and 25 minutes post bolus can be seen in Table 3. At baseline, the LF/HF ratio was 0.75±0.25, indicating that subjects were mainly under parasympathetic control. Administration of pentolinium led to a significant drop in HF power (ANOVA: *P*=0.001), LF power (*P*=0.01), and total power (*P*=0.001; Figure 2A) in comparison to placebo. After pentolinium MAP (Figure 2B), HR and CO all increased (ANOVA: *P*=0.004, *P*=0.04, and *P*=0.005, respectively) and SV fell (*P*=0.002) in comparison to placebo. There was no statistically significant difference in the change in aPWV between saline and pentolinium (*P*=0.1; Figure 2C).

**Table 3.**
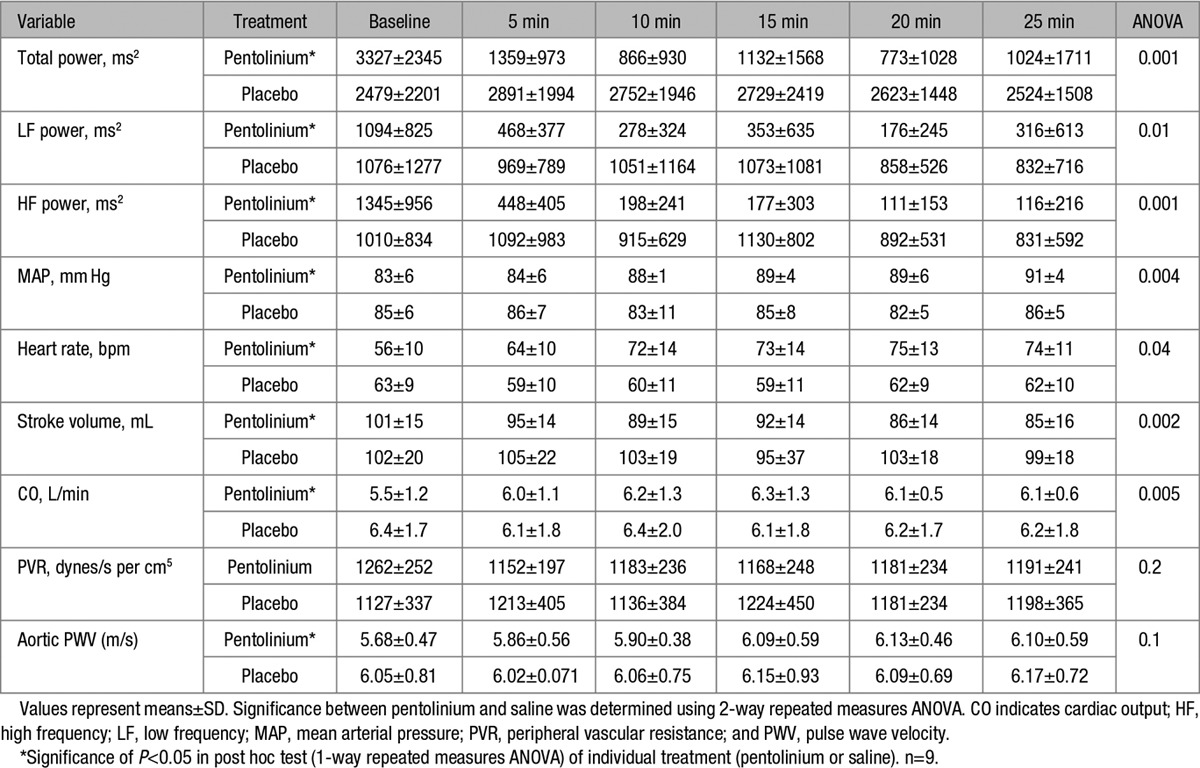
The Effect of Pentolinium and Saline on Heart Rate Variability and Hemodynamics

### Study 3

A total of 12 subjects were studied. The mean age of the subjects was 31±6 years, and 6 were female and 6 were male. The mean BMI was 23.1±2.9 kg/m^2^ and the mean BP was 115±11/69±8 mm Hg. Detailed hemodynamic and HRV changes at baseline and during handgrip exercise can be seen in Table 4. During the isometric handgrip exercise, LF/HF ratio increased from 1.32±0.91 to 2.09±1.21; *P*=0.003, indicating an activation of the SNS. MAP increased from 84±9 to 92±10 mm Hg; *P*<0.0001, as did aPWV (6.25±1.18 versus 6.67±1.26 m/s; *P*=0.013). However, when the change in aPWV was adjusted for the change in MAP, the effect was abolished (*P*=0.1).

**Table 4.**
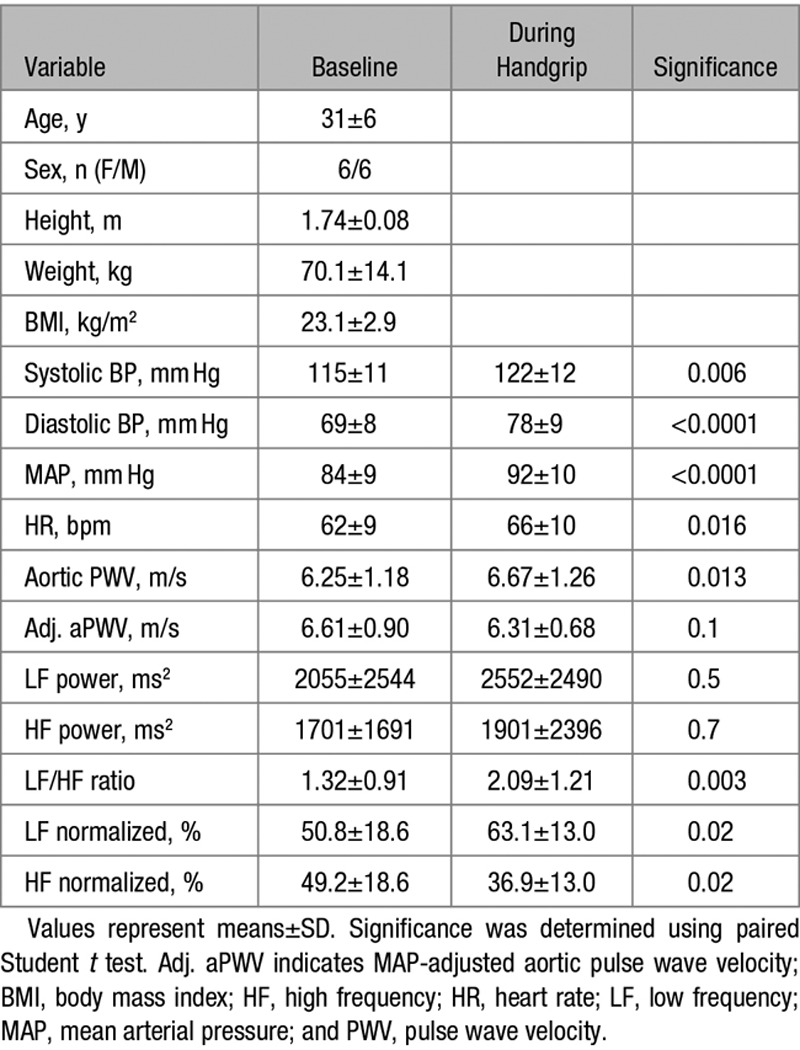
Demographics and the Effect of Isometric Handgrip Exercise on Hemodynamics and HR Variability

## Discussion

The main finding of the 3 studies presented is that the ANS does not influence aPWV independently of MAP and HR in young healthy individuals. In cross-sectional analyses, we found that aPWV was lowest in those subjects with the highest parasympathetic activity, but this association did not remain significant after adjusting for MAP and HR. Interestingly, when we divided the subjects according to the LF power, an index of sympathetic activity, we did not see any relationship with aPWV. Other cross-sectional studies investigating the role of the ANS in aortic stiffness have conflicting results. Our results are supported by a study in patients with kidney disease,^10^ showing that HRV is not associated with aPWV. However, they did not adjust aPWV for MAP. Jaiswal et al^9^ also concluded that aPWV is not associated with HRV in healthy subjects, but they did report an association in diabetic patients. However, they did not account for the influence of the parasympathetic nervous system (PNS) and SNS separately.^9^ Swierblewska et al^8^ reported that SNS activity was associated with aPWV in healthy subjects in a small study using muscle sympathetic neural activity (MSNA) as a measure of ANS activity. However, it is not clear how well MSNA reflects vascular SNS activity, and it does not assess PNS activity. A previous study showed that MSNA correlates inversely with total peripheral resistance, but not with MAP or DBP.^20^ Nevertheless, our data suggest that reduced parasympathetic tone may be an important regulator of BP in young subjects. Our findings are supported by a study in which young normotensive subjects with a family history of hypertension had reduced PNS activity and higher BP, but similar SNS activity compared with matched subjects without a family history of hypertension.^21^ Julius et al^22^ concluded that not all borderline hypertension can be accounted by increased SNS activity, but that reduced parasympathetic tone plays an important role at least in young individuals. To our knowledge, this study is the first to examine at the role of both PNS and SNS activity in aortic stiffness in a large cohort of young healthy individuals, adjusting for the confounding influence of MAP and HR. Arterial stiffness measurements are confounded by distending pressure; and therefore, it is important to compute PWV at a similar MAP, that is, isobaric stiffness. Our cross-sectional data clearly demonstrate that differences in aPWV disappear after adjusting for MAP.

Numerous studies have demonstrated that SNS activation regulates the smooth muscle tone in muscular arteries. Failla et al^23^ showed that radial artery distensibility increased by ipsilateral anesthesia of the brachial plexus, and similarly, femoral artery distensibility was increased by both ipsilateral subarachnoid anesthesia and ipsilateral sympathetic gangliectomy, whereas no effect was seen in the contralateral arteries. Pannier et al^24^ used lower body negative pressure to stimulate the SNS and demonstrated that it led to a reduction of carotid artery distensibility. More recently, HRV parameters have been associated with brachial-ankle PWV.^25^ However, the human arterial tree has a heterogeneous structure with the relative amount of smooth muscle continuously increasing and the elastin:collagen ratio decreasing toward the periphery.^26^ Therefore, whether the smooth muscle cells in the elastic aorta have a contractile role to dynamically modify aortic stiffness is highly contentious, and in vitro experiments in human aorta have not shown any regulatory effect on aortic smooth muscle tone.^7^ Furthermore, aortic stiffness, but not muscular artery stiffness, has predictive value,^27^ which is why aortic PWV is considered as the gold-standard measure of arterial stiffness.

Our 2 interventional studies modified ANS activity to gain a better understanding of the causal relationship between ANS activity and aortic stiffening. As expected, pentolinium markedly reduced ANS activity, leading to an increase in HR, MAP, and CO, but there was no change in aPWV in comparison to placebo. As pentolinium is used for the treatment of hypertensive crises,^28^ our results may seem unexpected. However, in young people, parasympathetic tone is greater than sympathetic tone when lying down (as demonstrated by our baseline LF/HF ratio of 0.75±0.25), and there is a net suppression of intrinsic HR, that is, the SA node wants to fire more quickly. As pentolinium blocks both the PNS and SNS HR increases with the removal of parasympathetic innervation of the SA node. This is contrary to what we may have seen in an older or hypertensive population who tend to be sympathetically driven at rest.^2^ To produce the opposite effect, we used isometric handgrip exercise to activate the SNS. LF/HF ratio increased and was accompanied by a rise in HR, MAP, and aPWV. However, after adjusting for changes in MAP, the increase in aPWV was abolished. These results support the findings from our cross-sectional study, highlighting that the ANS does not have a direct BP-independent effect on aortic stiffness. To our knowledge, this is the first study to investigate the effect of ganglion blockade on aortic stiffness in young, healthy subjects. A previous study in hypertensive patients demonstrated that combined α–β-receptor blockade induced by labetalol or propranolol and phentolamine reduced BP and aortic stiffness, but this was not adjusted for BP,^13^ making it difficult to dissect the direct effect on aortic stiffness. Previous studies demonstrate that isometric handgrip increases SNS activity^19^ and forearm blood flow,^29^ but there are only a few human studies that have examined at the effect of SNS activation on aortic stiffness. Sonesson et al^11^ showed that lower body negative pressure-induced SNS activation, but did not alter the aortic wall distensibility. On the contrary, a recent study reported that aPWV increased in patients with CAD during isometric handgrip exercise; however, the authors did not adjust for the change in MAP.^12^

### Potential Limitations

Our study has some limitations. We assessed the ANS activity using an indirect measure. MSNA is often considered to be the gold standard measurement of SNS activity, but it is important to recognize that there are regional differences in the ANS activity and that muscle ANS activity does not necessarily reflect activity in the vasomotor nerves innervating the aorta, and indeed, MSNA does not correlate with MAP in young men.^20^ Furthermore, MSNA does not provide information on PNS activity. This may be important in young individuals when PNS activity can dominate. Indeed, we did find that changes in HRV parameters were closely related to changes in HR and MAP after intervention suggesting that HRV is a reliable measure of the ANS activity of the cardiovascular system. We deliberately chose to study young individuals to minimize the influence of atherosclerosis and arteriosclerosis, but we cannot, therefore, extrapolate our findings to older individuals or those with increased SNS activity or hypertension. Another limitation of our study was the fact that HRV does not assess SNS activity per se, as LF power represents both the activity of SNS and PNS, whereas HF power represents mainly PNS activity. However, previous data suggest that MSNA correlates well with LF power, but only when subjects’ sympathetic activity is increased.^30^ Finally, we cannot exclude a long-term influence of SNS activation on aortic stiffness.

### Perspectives

We have demonstrated in young healthy subjects that aortic stiffness is not directly regulated by the ANS, but does change in response to changes in HR and MAP. Although the ANS does not have a direct role in the regulation of aortic stiffness, it is apparent that reduced parasympathetic tone has a profound effect on the control of BP in young individuals, which subsequently will lead to a secondary increase in aPWV. Aortic PWV, in turn, is a powerful, independent predictor of future cardiovascular events and all-cause mortality. Therefore, a simple 5-minute recording of HRV could provide useful additive information for clinicians when choosing the ideal antihypertensive drugs for young hypertensives. β-adrenergic antagonists and ACE inhibitors, which increase PNS and reduce SNS activity, could be favorable in young patients with reduced parasympathetic activity.^31^ In addition, simple lifestyle modifications leading to weight loss increase cardiac vagal drive and reduce sympathetic nerve activity and BP in young subjects.^32^ We have identified some possible early mechanisms for hypertension and aortic stiffening in young people, and this may be useful in targeting drug interventions and developing novel therapeutics for the treatment of hypertension in the young subjects.

## Sources of Funding

I.B. Wilkinson, S.M.L. Barrett, and C.M. McEniery were funded by British Heart Foundation. K.M. Mäki-Petäjä, I.B. Wilkinson, C.M. McEniery, and J. Cheriyan received funding from the Comprehensive Local Research Network and from the National Institute for Health Research: Cambridge Comprehensive Biomedical Research Centre.

## Disclosures

None.

## Novelty and Significance

### What Is New?

To our knowledge, this study is the first to look at the role of both parasympathetic and sympathetic nervous system activity in aortic stiffness in large cohort of young healthy individuals, adjusting for confounding factors of mean arterial pressure and heart rate (HR). Our key findings were:

- Aortic stiffness is highest in subjects with the lowest parasympathetic activity.
- The autonomic nervous system does not have a pressure-independent role in regulating aortic stiffness via changes in smooth muscle tone. Instead, aortic stiffening is driven by changes in HR and mean arterial pressure.
- Stimulation of the sympathetic nervous system leads to increases in HR and blood pressure resulting in aortic stiffening.

### What Is Relevant?

- A reduced parasympathetic tone has a profound effect on the control of blood pressure and subsequent aortic stiffening in young individuals.
- A simple 5-minute recording of HR variability could provide useful additive information for clinicians when choosing the ideal antihypertensive treatment for young hypertensives.

### Summary

The main finding of the present series of studies is that in young healthy subjects, the autonomic nervous system regulates aortic stiffness via changes in HR and mean arterial pressure.
